# Characterization of Modular Bacteriophage Endolysins from *Myoviridae* Phages OBP, 201ϕ2-1 and PVP-SE1

**DOI:** 10.1371/journal.pone.0036991

**Published:** 2012-05-15

**Authors:** Maarten Walmagh, Yves Briers, Silvio Branco dos Santos, Joana Azeredo, Rob Lavigne

**Affiliations:** 1 Laboratory of Gene Technology, Katholieke Universiteit Leuven, Leuven, Belgium; 2 Institute of Food, Nutrition and Health, ETH Zurich, Zurich, Switzerland; 3 IBB - Institute for Biotechnology and Bioengineering, Centre of Biological Engineering, Universidade do Minho, Braga, Portugal; Research Center Borstel, Germany

## Abstract

Peptidoglycan lytic enzymes (endolysins) induce bacterial host cell lysis in the late phase of the lytic bacteriophage replication cycle. Endolysins OBPgp279 (from *Pseudomonas fluorescens* phage OBP), PVP-SE1gp146 (*Salmonella enterica* serovar Enteritidis phage PVP-SE1) and 201ϕ2-1gp229 (*Pseudomonas chlororaphis* phage 201ϕ2-1) all possess a modular structure with an N-terminal cell wall binding domain and a C-terminal catalytic domain, a unique property for endolysins with a Gram-negative background. All three modular endolysins showed strong muralytic activity on the peptidoglycan of a broad range of Gram-negative bacteria, partly due to the presence of the cell wall binding domain. In the case of PVP-SE1gp146, this domain shows a binding affinity for *Salmonella* peptidoglycan that falls within the range of typical cell adhesion molecules (K_aff_ = 1.26×10^6^ M^−1^). Remarkably, PVP-SE1gp146 turns out to be thermoresistant up to temperatures of 90°C, making it a potential candidate as antibacterial component in hurdle technology for food preservation. OBPgp279, on the other hand, is suggested to intrinsically destabilize the outer membrane of *Pseudomonas* species, thereby gaining access to their peptidoglycan and exerts an antibacterial activity of 1 logarithmic unit reduction. Addition of 0.5 mM EDTA significantly increases the antibacterial activity of the three modular endolysins up to 2–3 logarithmic units reduction. This research work offers perspectives towards elucidation of the structural differences explaining the unique biochemical and antibacterial properties of OBPgp279, PVP-SE1gp146 and 201ϕ2-1gp229. Furthermore, these endolysins extensively enlarge the pool of potential antibacterial compounds used against multi-drug resistant Gram-negative bacterial infections.

## Introduction

The recent genome analysis of the *Myoviridae* bacteriophages *Pseudomonas fluorescens* OBP [1] and *Pseudomonas chlororaphis* 201ϕ2-1 [2], both member of the “phiKZ-like viruses”, and the *Salmonella enterica* serovar Enteritidis (further abbreviated as *S.* Enteritidis) PVP-SE1, a “rV5-like virus” [3] reveals an abundance of interesting enzymes and functions. Among those are the predicted peptidoglycan (PG) degrading hydrolases (endolysins) OBP gene product (gp) 279 (YP_004958186.1) [1], 201ϕ2-1gp229 (YP_001956952.1) [2] and PVP-SE1gp146 (YP_004893953.1) [3] involved in bacterial host lysis and progeny release at the end of the phage lytic replication cycle.

The use of externally applied endolysins from phages infecting Gram-positive bacteria as antibacterial compounds has proven successful in various applications [4]. However, the use of endolysins from Gram-negative origin against Gram-negative pathogens is an unexploited research focus as these bacteria possess an outer membrane (OM) leaflet which prevents efficient peptidoglycan lysis [5].

In this context, *Pseudomonas aeruginosa* phage endolysins KZ144 (from giant phage ϕKZ) and EL188 (giant phage EL) were recently shown to have lytic activity on the PG of *P. aeruginosa* PAO1 [6]. KZ144 and EL188 possess a modular structure composed of an N-terminal cell wall binding domain and a C-terminal catalytic domain which is rare amongst endolysins from Gram-negative origin [6], [7]. The presence of this cell wall binding domain is linked with the high muralytic activity of KZ144 and EL188 on OM permeabilized *P. aeruginosa* [6].

In this paper we expand the pool of modular endolysins from Gram-negative infecting *Myoviridae* phages with three new candidates (OBPgp279, 201ϕ2-1gp229 and PVP-SE1gp146) and extensively describe and compare them at the biochemical and antibacterial level. These data help to elucidate their fundamental properties and emphasize their potential as antibacterial agents towards treatment of Gram-negative infections.

## Materials and Methods

### Bacterial strains

*P. aeruginosa* PAO1 from two different sources was used in this study: a PAO1 strain donated by prof. V. Krylov (Institute of Genetics and Selection of Industrial Microorganisms in Moscow, Russia) for the biochemical characterization and peptidoglycan binding assay, and a PAO1 strain from Pirnay [8] for the *in vitro* antibacterial assay. Both strains only differ in pili formation, a difference which has no further impact on the obtained results. For this reason, we consistently named the strain PAO1 throughout the manuscript. The clinical *P. aeruginosa* Br667 strain was a burn wound isolate from the Military Queen Astrid Hospital (Brussels, Belgium) that is multidrug-resistant against 10 out of 11 tested antibiotics due to reduced OM permeability [8]. *Escherichia coli* XL1-Blue and *E. coli* BL21(DE3)pLysS are commercially available expression strains (Agilent Technologies, Santa Clara, CA, USA). *Salmonella enterica* serovar Typhimurium LT2 (further abbreviated as *S.* Typhimurium LT2), *Staphylococcus aureus* subsp. *aureus* Rosenbach ATCC 6538, *Micrococcus lysodeikticus* ATCC 4698, *Bacillus subtilis* PSB3 and *Lactococcus lactis* subsp. *lactis* were kindly provided by the Centre of Food and Microbial Technology (University of Leuven, Belgium). All these strains were grown at 37°C in Lysogeny Broth (LB).

### Isolation and cloning of endolysin open reading frames and derivatives

Open reading frame (ORF) 279 from phage OBP, ORF 229 from phage 201ϕ2-1, ORF 146 of PVP-SE1 and the sequences encoding the individual catalytic domains of each endolysin were all amplified (Pfu polymerase, ThermoFischer Scientific, Waltham, MA, USA, Table S1) using genomic DNA template isolated from the respective phages. All obtained PCR products were cloned into the pEXP5CT-TOPO® expression vector according to the manufacturer's manual (Life Technologies, Carlsbad, CA, USA) and sequence-verified (Big Dye polymerase, ABI3130 Sequencher, Life Technologies). The pEXP5CT-TOPO® vector provides a C-terminal His_6_-tag for efficient Ni^2+^- NTA purification.

To obtain fusion proteins of the peptidoglycan binding domains (PBD) of endolysins OBPgp279, PVP-SE1gp146 and 201ϕ2-1gp229 to the *Enhanced Green Fluorescent Protein* (EGFP), the corresponding sequences encoding the different PG binding moieties of the endolysins were amplified (Pfu polymerase, Table S1) and spliced to the EGFP encoding sequence (Takara, Otsu, Japan) using a *Bam*HI restriction site. The fusion fragments were cloned into the pEXP5CT-TOPO® expression vector for protein expression and purification.

### Large scale expression and purification of endolysins and their derivatives

All proteins were expressed for 4 hours at 37°C or for 18 hours at 16°C in *E. coli* BL21(DE3)pLysS cells (Life Technologies) upon induction of mid-exponential growing cells (optical density at 600 nm wavelength (OD_600 nm_) of 0.6) using 1 mM of isopropyl-β-D-thiogalactopyranoside (IPTG). After expression, the bacterial cell pellet was resuspended in a lysis buffer (0.5 M NaCl, 20 mM of NaH_2_PO_4_/NaOH, pH 7.4), and disrupted with a combination of freeze-thawing (−80°C/room temperature) and sonication (10 cycles of 30 s pulse and 30 s rest, Vibra-Cell™ Sonics and Materials, Newtown, CT, USA). For efficient lysis of expression strains producing the EGFP fusion proteins, 10 mg Hen Egg white Lysozyme (HEWL, Across Organics, Belgium) was added to the lysis buffer. Recombinant endolysin was purified on Ni^2+^-NTA resin stacked HisTrap™ HP 1 ml columns (GE Healthcare, Waukesha, WI, USA) using protein-dependent imidazole concentrations in the washing buffer (0.5 M NaCl, 20 mM of NaH_2_PO_4_/NaOH, 60–70 mM imidazole, pH 7.4) (Table S1). Purity of the recombinant proteins was estimated to be more than 90% by SDS-PAGE. Protein concentrations were determined spectrophotometrically after dialyzing against a phosphate buffered saline (PBS) pH 7.4 buffer using Slide-A-Lyzer® MINI dialysis units (ThermoFischer Scientific, Table S1).

### Peptidoglycan binding capacity assay

The binding capacity of the PBDs in OBPgp279, PVP-SE1gp146 and 201ϕ2-1gp229 was experimentally verified using a fluorescence assay modified for Gram-negative cell walls [6]. For this assay, *P. aeruginosa* PAO1 or *S.* Typhimurium LT2 cells with permeabilized OM are used, according to [9]. Summarized, mid-exponentially growing *P. aeruginosa* PAO1 or *S.* Typhimurium LT2 cells (OD_600 nm_ = 0.6) were spun down and subsequently incubated for 45 min in chloroform-saturated 0.05 M Tris/HCl (pH 7.7) at room temperature. Subsequently, cells were washed in a KH_2_PO_4_/K_2_HPO_4_ buffer (ionic strength of 80 mM, pH 7.2) to remove residual chloroform and concentrated to an OD_600 nm_ of 1.5. The fusion proteins OBP_1–117_-EGFP, OBP_7–54_-EGFP, OBP_57–117_-EGFP, PVP_1–63_-EGFP, 201ϕ2-1_8–63_-EGFP and the wild type (wt) EGFP (negative control) were incubated at a concentration of 2 µM with the OM permeabilized *P. aeruginosa* PAO1 and *S.* Typhimurium LT2 cells, in a total volume of 200 µl for 5 min at room temperature. Unbound fusion proteins were removed with two consecutive washing steps in 5 mM HEPES/NaOH buffer (pH 7.2). Cells were visualized using epifluorescence microscopy (Leica type DMLB DS 0.6.23./100 S/F, McBain instruments, Chatsworth, NJ, USA, equipped with a LED light source Fluo LED 4000, McBain Instruments and a GFP filter). At conditions where no binding occurred, epifluorescence was overlaid by phase contrast to visualize uncolored cells.

To quantify binding, cell suspensions (100 µl) were incubated with 2 µM fusion protein in a total volume of 200 µl. Subsequently, the labeled cells were washed twice with 5 mM HEPES/NaOH buffer (pH 7.2), transferred to microplate wells and placed in the Fluoroskan Ascent FL fluorescence reader (ThermoFischer Scientific). Fluorescence was measured at 520 nm, using an excitation wavelength of 480 nm. The OD_600 nm_ was measured in parallel and fluorescence was expressed per unit of OD_600 nm_ to take small differences in cell density into account.

### Peptidoglycan binding affinity measured by surface plasmon resonance

Surface plasmon resonance (BIAcore X, BIAcore AB, Uppsala, Sweden) was used to obtain sensorgrams for binding of the fusion protein PVP_1–63_-EGFP to the surface of immobilized, autoclaved *S.* Typhimurium LT2 cells [10]. In a first step, the carboxymethylated surface of a BIAcore Pioneer C1 chip was coated with 0.5 mg/ml PVP_1–63_-EGFP (flow rate = 5 µl/min; 10 mM sodium acetate pH 5.0), using the amine coupling technique. Ethanolamine was added to block unbound positions on the chip. Secondly, autoclaved *S.* Typhimurium LT2 cells (density of 3×10^10^ cells/ml) resuspended in HBS-T buffer (10 mM HEPES, 150 mM NaCl, 3.4 mM EDTA, 0.005% Tween20, pH 7.8) were applied to the chip surface containing covalently bound PVP_1–63_-EGFP (total volume = 30 µl, flow rate = 3 µl/min). The obtained double layer served as the basis for the binding affinity measurements, performed at 25°C with 2 different concentrations (5 and 10 µM) of PVP_1–63_-EGFP for 3 min (association phase) and 12 min (dissociation phase) (flow rate = 10 µl/min). Each concentration was measured in three-fold. Regeneration of the coated chip in between different measurements takes place by several washing steps with increasing NaCl concentrations (2 M, 3 M, 4 M; 50 µl; 10 µl/min). Kinetic data are calculated based on the measurements using the BIA-evaluation software (version 3.0, BIAcore).

### Quantification and characterization of muralytic activity

The PG lytic (or muralytic) activity of the purified proteins and their catalytic domains were quantified on *P. aeruginosa* PAO1, *S.* Typhimurium LT2 and *E. coli* XL1-Blue cells with a chloroform/Tris permeabilized OM [9]. In this assay, 30 µl of enzyme was added to 270 µl of OM permeabilized cells, resulting in a decrease of the optical density over time, as measured spectrophotometrically at 655 nm using a Microplate Reader 680 (Bio-Rad, CA, USA). A standardized calculation method to quantify the muralytic activities of lytic enzymes using this assay has been optimized and extensively described [11]. Obtained values for the negative control (30 µl of PBS pH 7.4) were subtracted from the sample values.

The influence of pH on the muralytic activity was assessed using OM permeabilized cells resuspended in an universal pH buffer (150 mM KCl, 10 mM KH_2_PO_4_, 10 mM Na-citrate and 10 mM H_3_BO_4_) adjusted to different pH's between 3 and 10. To investigate the thermal stability of OBPgp279, 201ϕ2-1gp229 and PVP-SE1gp146 after heat treatment, 5 µM of each endolysin was incubated at 42°C and 50°C for different time-intervals (1, 2, 3, 4, 8 and 24 hours) in a Biometra T3000 Thermocycler (Göttingen, Germany) followed by a cooling step to room temperature. Proteins showing full activity at 50°C were heated up to 60, 70, 80, 90 and 100°C during 20, 40 and 60 min time-intervals and cooled down again. The residual muralytic activity of each sample relative to the activity of unheated reference sample at time 0 ( = 100% activity) was determined.

### *In vitro* antibacterial activity assay

Mid-exponentially growing *P. aeruginosa* PAO1, *P. aeruginosa* Br667, *E. coli* XL1-Blue and *S.* Typhimurium LT2 cells (OD_600 nm_ = 0.6) were 100-fold diluted in 5 mM HEPES/NaOH (pH 7.2) to a final density of 10^6^ colony forming units/ml. Each cell culture (100 µl) was incubated for 30 min at room temperature with 50 µl OBPgp279 (1.5 µM final concentration), PVP-SE1gp146 (5 µM) or 201ϕ2-1gp229 (3 µM) dialyzed against a PBS buffer (pH 7.4) together with 50 µl of 5 mM HEPES/NaOH (pH 7.2) buffer or 50 µl of EDTA (final concentration of 0.5 mM) dissolved in the same buffer. As negative control, 100 µl of cells were added to 50 µl of 5 mM HEPES/NaOH (pH 7.2) and 50 µl of a PBS buffer (pH 7.4). After incubation, cell suspensions were diluted in three-fold to 10^5^, 10^4^ and 10^3^ cells/ml and 100 µl of each dilution was plated out on LB medium. Colonies were counted after an overnight incubation at 37°C. The antibacterial activity was quantified as the relative inactivation in logarithmic units ( = log_10_(N_0_/N_i_) with N_0_ = number of untreated cells (in the negative control) and N_i_ = number of treated cells counted after incubation).

## Results

### Predicted modular domain structures of OBPgp279, PVP-SE1gp146 and 201ϕ2-1gp229

**Figure 1:**
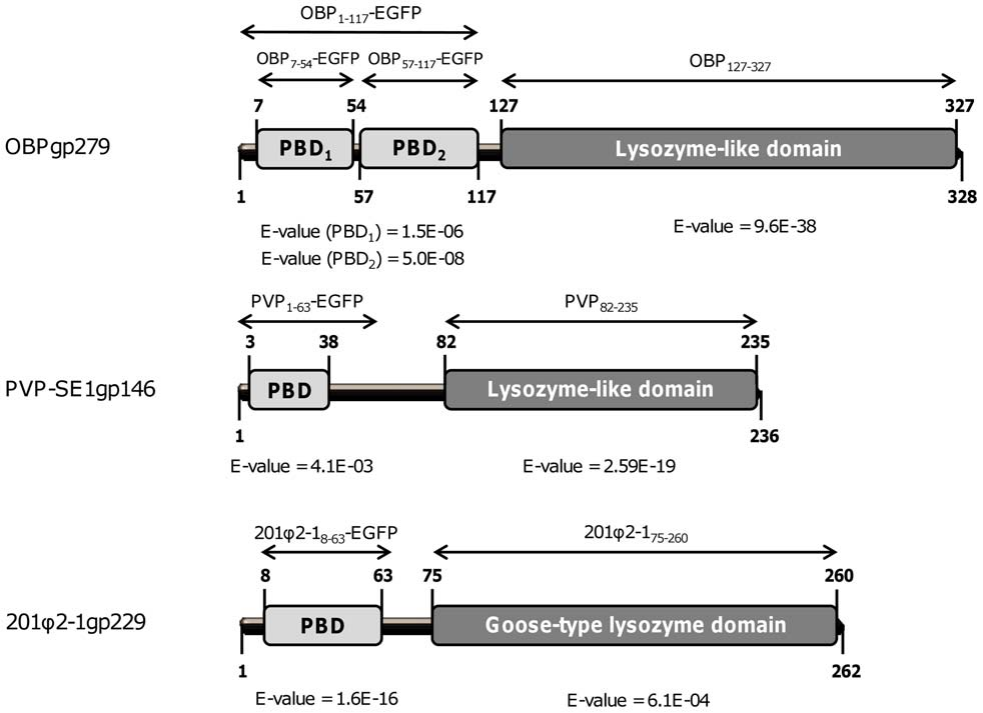
Schematic diagram of the domain organization of OBPgp279, 201ϕ2-1gp229 and PVP-SE1gp146. For each endolysin, the predicted N-terminal PG binding domains (PBD) are marked in light grey, whereas the C-terminal catalytic domains (lysozyme-like or goose-like lysozyme domains) are in dark grey. E-values for OBP_127–327_ (predicted by HHpred), PBD_1_ and PBD_2_ of OBPgp279 (both HHpred), PVP_82–235_ (Blastp), PBD of PVP-SE1gp146 (Pfam), 201ϕ2-1_75–210_ (HHpred) and PBD of 201ϕ2-1gp229 (Pfam) are all indicated beneath the respective domains. The names of the different constructs used in this study are also marked above each predicted structure.

Using Blastp, Pfam and HHpred prediction tools [12]–[14], endolysins OBPgp279 (from *P. fluorescens* infecting phage OBP), 201ϕ2-1gp229 (from *P. chlororaphis* infecting phage 201ϕ2-1) and PVP-SE1gp146 (from *S.* Enteritidis infecting phage PVP-SE1) were predicted to adopt a modular structure with an N-terminal PBD and a C-terminal catalytic domain (Figure 1). The catalytic domain of 201ϕ2-1gp229 showed similarity with a goose-type lysozyme domain, known for its three-dimensional structural correspondence with bacteriophage T4 lysozyme and HEWL [15] (Figure 1). The catalytic domains of OBPgp279 and PVP-SE1gp146 both contained a conserved sequence motif (Figure S1), which was typically found in members of the glycoside hydrolase 19 (GH19) family (general sequence motif = F/H/Y-G-R-G-A/P-X-Q-I/L-S/T-F/H/Y/W-H/N-F/Y-N/Y, X = hydrophilic amino acid) [16]. Other recently characterized endolysins like the *Acinetobacter baumannii* phage ϕAB2 lysin (LysAB2, ADX62345.1) [17] or the *Salmonella enterica* phage ε15 lysin (NP_848233.1) [18] also belong to this GH19 family. Both the goose-type lysozyme and the GH19 family are members of the lysozyme-like superfamily which contains a broad range of lytic domains involved in hydrolysis of the β-1,4 linkages between the N-acetylmuramine and N-acetylglucosamine sugars of the PG backbone [19].

The N-terminal domains of OBPgp279, 201ϕ2-1gp229 and PVP-SE1gp146 possess specific repeated motifs which are conserved in the confirmed PBDs of the modular endolysins KZ144 and EL188 [6]. These repeated motifs largely matched the consensus sequence (D-G-(Pho)_2_-G-K/N-G/N-T, Pho = hydrophobic amino acid) proposed previously [6]. Two such motifs (D-G-L-F-G-E-K-C and D-G-K-W-G-G-T) were present in the N-terminal domain of OBPgp279 (one in each predicted sub-domain) and one in 201ϕ2-1gp229 (D-G-V-F-G-D-N-T) and PVP-SE1gp146 (D-G-L-Y-G-P-A-T) (Figure S1). This conserved feature is seen in many proteins that interact with repeated structures such as PG [20]. For OBPgp279, seven additional amino acids (L-A-X-Pho-X-L-Y, X = same hydrophilic amino acid, Pho = hydrophobic amino acid) were detected in front of both repeated motifs with four amino acids in between, strengthening the idea that the PBDs of OBPgp279 were duplicated throughout evolution. The presence of these PG binding motifs inside the N-terminal domains strongly supported the predicted PG binding function. Furthermore, the repeated motif observation suggested the presence of a general sequence conserved among all PBDs of modular endolysins with a Gram-negative background.

### N-terminal cell wall binding domains of OBPgp279, PVP-SE1gp146 and 201ϕ2-1gp229

#### Peptidoglycan binding capacity

**Figure 2:**
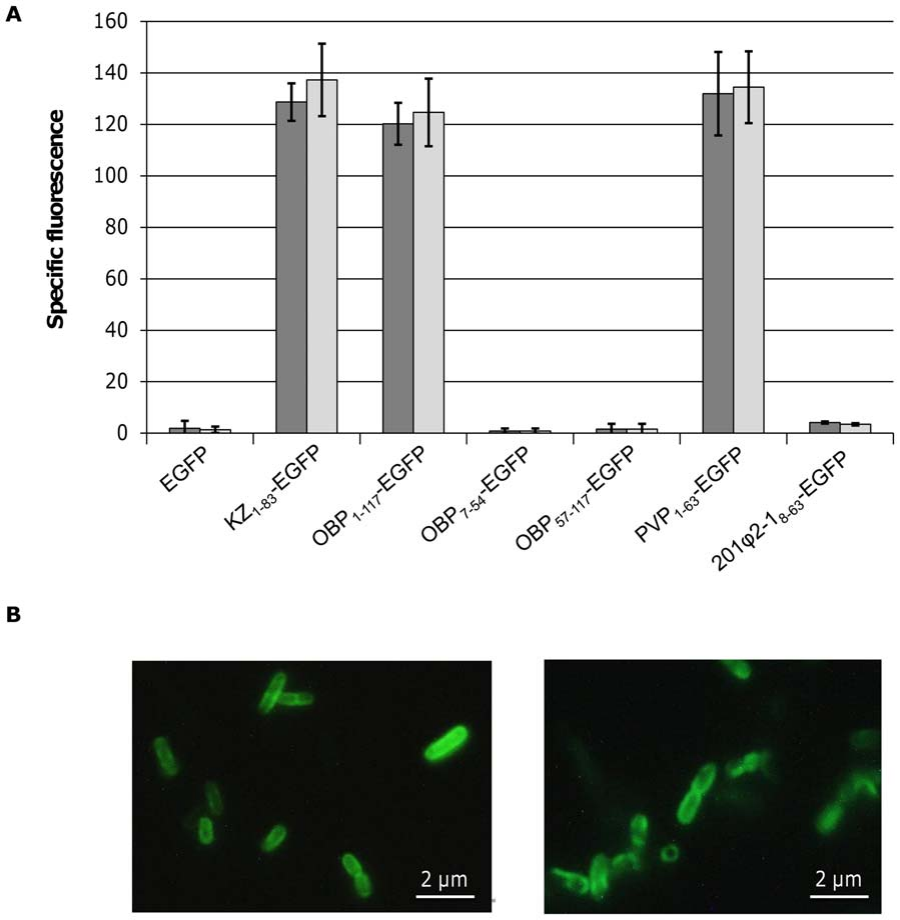
Peptidoglycan binding capacity of fusion proteins OBP_1–117_-EGFP, OBP_7–54_-EGFP, OBP_57–117_-EGFP, PVP_1–63_-EGFP and 201ϕ2-1_8–63_-EGFP. (A) The specific fluorescence (i.e. the fluorescence of the treated cells subtracted by the fluorescence of untreated cells and cell buffer) of 2 µM of each fusion protein measured after incubation with OM permeabilized *P. aeruginosa* PAO1 (dark grey bars) or *S.* Typhimurium LT2 cells (light grey bars) is shown here for the different EGFP fusion constructs. Obtained values were compared with the specific fluorescence of the unbound wt EGFP. 2 µM of KZ-EGFP, the fusion protein of the PBD of KZ144 and EGFP, was used as positive control. Average and standard deviation values of three independent experiments are shown. (B) Epifluorescence microscopy of OM permeabilized *P. aeruginosa* PAO1 cells treated with OBP_1–117_-EGFP (left) and PVP_1–63_-EGFP (right). Cells were incubated with 2 µM of each fusion protein for 5 min. Cell pellets were washed twice and visualized using epifluorescence microscopy (with 500× magnification, size bar is indicated). Both fusion proteins are visualized in green targeted to the bacterial cell wall.

To evaluate the PG binding capacity, we produced the fusion proteins OBP_1–117_-EGFP, PVP_1–63_-EGFP and 201ϕ2-1_8–63_-EGFP comprising the predicted binding domains of OBPgp279 (amino acids 3 to 117), PVP-SE1gp146 (amino acids 3 to 38) and 201ϕ2-1gp229 (amino acids 8 to 63), N-terminally fused to EGFP. For this purpose, OM permeabilized *P. aeruginosa* PAO1 and *S.* Typhimurium LT2 cells [9] were incubated with 2 µM fusion protein. Cells were washed to remove unbound protein and the retained fluorescence of PG bound protein was quantified (Figure 2A). A negative wt EGFP control and a positive KZ_1–83_-EGFP [6] control were included for comparison. The results showed that the N-terminal domains of both OBPgp279 and PVP-SE1gp146 have PG binding activity, since the measured fluorescence levels under saturating concentrations were comparable with KZ_1–83_-EGFP [6]. This observation also indicated that the same number of binding sites was occupied by the fusion proteins. For 201ϕ2-1_8–63_-EGFP, no fluorescence was detected, possibly due to improper folding of the fusion protein during expression. Binding capacity of OBP_1–117_-EGFP and PVP_1–63_-EGFP was visually confirmed using epifluorescence microscopy (Figure 2B). The cell wall of the targeted *P. aeruginosa* PAO1 and *S.* Typhimurium LT2 cells became fluorescent in less than 5 min after incubation with the fusion constructs OBP_1–117_-EGFP and PVP_1–63_-EGFP. However, a single subdomain (either OBP_7–54_ or OBP_57–117_) of OBPgp279 was not sufficient for cell wall binding since no fluorescence was retained with any of both fusion proteins (Figure 2A). This could be explained because of improper folding of the single subdomains in the absence of the other subdomain, or because both subdomains were needed simultaneously for effective PG binding.

#### Peptidoglycan binding affinity of PVP_1–63_-EGFP

For PVP_1–63_-EGFP, the interaction strength with the PG of *S.* Typhimurium LT2 ( = ligand) was analyzed using surface plasmon resonance analysis [10]. This technique made it possible to follow the interaction between the fusion proteins and immobilized Gram-negative cells in real time and to determine the binding affinity constant of each interaction. For this, autoclaved *S.* Typhimurium LT2 cells were immobilized on a PVP_1–63_-EGFP coated sensor chip. To this double layer, free PVP_1–63_-EGFP proteins were added allowing us to observe the interaction between the fusion proteins and the immobilized cells.

To calculate the apparent affinity constant *K_aff_* for binding of the fusion proteins to the Gram-negative cell wall, we used an indicative two-state model which assumes a 1∶1 binding (AB) between fusion protein (A) and ligand (B) occurs followed by a conformational change in the complex (AB^..^):
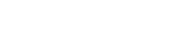
This proposed model enabled us to calculate the association (*k*_a1_), dissociation (*k*_d1_), forward (*k*_a2_) and backward (*k*_d2_) rate constants from which a *K*_aff_ constant of 1.26×10^6^ M^−1^ (or a dissociation constant of 7.93×10^−7^ M) was determined for fusion protein PVP_1–63_-EGFP (Table 1). The PG binding affinity constant of the PBD of PVP-SE1gp146 fell within the range of average SH2 domains and typical cell adhesion molecules [21].

**Table 1:** Apparent association and dissociation kinetic data for the binding of PVP_1–63_-EGFP to immobilized *S.* Typhimurium LT2 cells.

Analyte concentration		A+B↔AB		AB↔AB^..^		
(M)	(µg ml^−1^)	Association rate constant	Dissociation rate constant	Forward rate constant	Backward rate constant	Apparent affinity constant
		k_a1_ (M^−1^ s^−1^)	k_d1_ (s^−1^)	k_a2_ (s^−1^)	k_d2_ (s^−1^)	(M^−1^)
**5.00×10^−6^**	176	6.78×10^3^	3.34×10^−2^	9.64×10^−2^	2.25×10^−3^	1.09×10^6^
**10.0×10^−6^**	352	1.14×10^3^	5.94×10^−2^	3.94×10^−3^	6.07×10^−4^	1.43×10^6^
**Average**						1.26×10^6^

Two different concentrations (5 and 10 µM) were measured in three-fold and the corresponding average is represented. The association rate (k_a1_), dissociation rate (k_d1_), forward rate (k_a2_) and backward rate (k_d2_) constants are calculated according to a two-state model that describes a 1∶1 binding (AB) of the fusion protein analyte (A) to the immobilized ligand (B), followed by a conformational change in the complex (AB→AB^..^).

### Biochemical properties of OBPgp279, 201ϕ2-1gp229 and PVP-SE1gp146

#### Substrate specificity

To confirm the predicted catalytic activity and to determine the bacterial host substrate spectrum, each endolysin (1 µM final concentration) was incubated with three different OM permeabilized Gram-negative cell substrates (*P. aeruginosa* PAO1, *S.* Typhimurium LT2 and *E. coli* XL1-Blue) and four intact Gram-positive bacteria with different structural types of peptidoglycan (*S. aureus* subsp. *aureus* Rosenbach ATCC 6538, *M. lysodeikticus* ATCC 4698, *B. subtilis* PSB3 and *L. lactis* subsp. *lactis*) (Figure S2). All three OM permeabilized Gram-negative cell substrates were efficiently lysed by the tested endolysins as shown by the decrease in OD, demonstrating their muralytic properties. In contrast, all tested Gram-positive bacteria survived the treatment even with an excess of OBPgp279, PVP-SE1gp146 and 201ϕ2-1gp229 (Figure S2).

#### Quantification of muralytic activity

The muralytic activities of OBPgp279, 201ϕ2-1gp229 and PVP-SE1gp146 were first quantified on OM permeabilized *P. aeruginosa* PAO1 as described previously [11]. At the optimal pH for enzymatic activity of 7.2 which corresponded to the pH of the bacterial cytoplasm (Figure S3), lysis of *P. aeruginosa* PAO1 could be detected upon addition of 10 to 20 nM endolysin. Enzymatic activity further increased linearly dependent on the endolysin concentration. A saturated enzymatic activity was reached at concentrations of 50, 200 and 100 nM for OBPgp279, 201ϕ2-1gp229 and PVP-SE1gp146, respectively (data not shown). Based on the linear relation between lysis and concentration, muralytic activity was expressed in enzymatic units (Table 2) [11]. OBPgp279 possessed a 1.5 to 6 times higher muralytic activity value than PVP-SE1gp146 and 201ϕ2-1gp229, respectively, and a 5 to 10 times higher value compared to the previously reported modular endolysins EL188 and KZ144 [6]. As expected, muralytic activity values determined on OM permeabilized *S.* Typhimurium LT2 and *E. coli* XL1-Blue were consistent with those obtained for *P. aeruginosa* PAO1 (data not shown).

**Table 2:** Comparison of muralytic activities of OBPgp279, PVP-SE1gp146, 201ϕ2-1gp229, the catalytic domains of OBPgp279 (OBP_127-327_) and of 201ϕ2-1 (201ϕ2-1_75–260_), with phage endolysins KZ144 and EL188.

	Muralytic activity (in units/mM)
**OBPgp279**	19,979,151 (0.983)
**PVPSE1gp146**	13,613,960 (0.992)
**OBP_127–327_**	12,361,837 (0.981)
**EL188**	*4,735,148 (0.983)
**201ϕ2-1gp229**	4,468,638 (0.995)
**KZ144**	*2,058,435 (0.992)
**201ϕ2-1_75–260_**	1,950,766 (0.970)

* [6] Briers *et al.*

The muralytic activities of the endolysins were calculated from the slope of the best linear regression of the corresponding saturation curves, according to the definition for enzyme unit adapted from Briers and coworkers [11]. OM permeabilized *P. aeruginosa* PAO1 cell substrate resuspended in the optimal KH_2_PO_4_/K_2_HPO_4_ buffer (pH 7.2) was used to test the enzymatic activity. The endolysins were dialyzed against a PBS buffer (pH 7.4). Activity values of KZ144 and EL188 [6] were marked with an asterisk. R-square values for each slope are indicated between brackets. Constructs were ranked from the highest to the lowest muralytic activity.

In addition, we tested the necessity of the peptidoglycan binding domain for proper muralytic activity. Therefore, the individual C-terminal catalytic domains of OBPgp279 (OBP_127–327_), PVP-SE1gp146 (PVP_82–235_) and 201ϕ2-1gp229 (201ϕ2-1_75–260_) were cloned and purified to compare their activity with the obtained value for the full length endolysin. In the case of PVP_82–235_, no soluble protein could be obtained despite changes in expression parameters. Expression of both OBP_127–327_ and 201ϕ2-1_75–260_ yielded pure protein in large amounts for activity testing (Table S1). In the absence of the PBD, the individual catalytic domains of OBPgp279 and 201ϕ2-1gp229 were still active, retaining 62 and 44% of the muralytic activity of the full length endolysins on OM permeabilized *P. aeruginosa* PAO1, respectively (Table 2). For OBP_127–327_, the residual value was significantly higher than the full length modular endolysins 201ϕ2-1gp229, KZ144 and EL188. These results indicated that both catalytic domains could work independently from the PBD part and emphasized that the PBD also had an important contribution to the total enzymatic activity of the full length modular endolysins.

#### Thermoresistance

**Figure 3:**
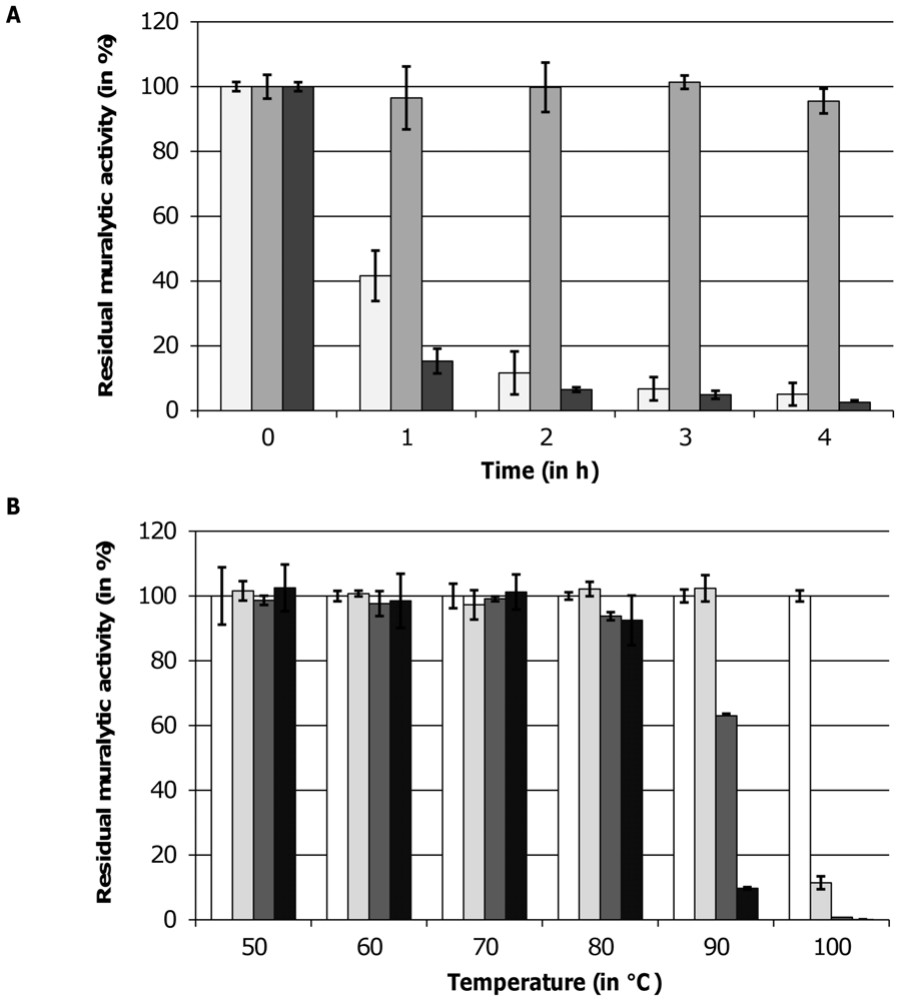
Thermoresistance of OBPgp279, PVP-SE1gp146 and 201ϕ2-1gp229. (A) The residual muralytic activity of OBPgp279 (1 µM, light grey bars), PVP-SE1gp146 (5 µM, intermediate grey bars) and 201ϕ2-1gp229 (3 µM, dark grey bars) on OM permeabilized *P. aeruginosa* PAO1 cell substrate after 1, 2, 3 and 4 h heat treatment at 50°C is shown. (B) For PVP-SE1gp146 (5 µM), the residual activity on OM permeabilized *P. aeruginosa* PAO1 after incubation for 0 (white bars), 20 (light grey bars), 40 (dark grey bars) and 60 (black bars) min on different temperatures between 50 and 100°C was determined. For each curve, averages and standard deviations of three repeated and independent experiments are given.

All three endolysins could be stored at 4°C in elution buffer for several weeks and months without loss of activity. The residual muralytic activity of each endolysin on OM permeabilized *P. aeruginosa* PAO1 was determined after a heat treatment at 42°C and 50°C and a subsequent cooling step to room temperature. No reduction of enzymatic activity was observed after prolonged incubation of the enzymes at 42°C (data not shown). When heated at 50°C, the muralytic activity of OBPgp279 and 201ϕ2-1gp229 dropped below 10% of the initial activity after 2 and 1 h incubation, respectively (Figure 3A). In contrast, PVP-SE1gp146 remained fully active, even after 24 h of incubation. At higher temperatures, PVP-SE1gp146 retained its maximal activity at 80°C incubation for 1 h and was only completely inactivated after 40 min at 100°C (Figure 3B).

### *In vitro* antibacterial activity with and without the OM permeabilizer EDTA

The antibacterial activity of OBPgp279, 201ϕ2-1gp229 and PVP-SE1gp146 was investigated on the Gram-negative bacteria *P. aeruginosa* PAO1, *P. aeruginosa* Br667, *E. coli* XL1-Blue and *S.* Typhimurium LT2, and compared with endolysins KZ144 and EL188 (Table 3A). To overcome the OM barrier, 0.5 mM EDTA was added (Table 3B). EDTA is extensively described as an effective OM permeabilizer [22], [23] and was shown to promote the penetration of endolysin EL188 from *P. aeruginosa* infecting phage EL through the OM of clinical *P. aeruginosa* isolates in mM concentrations [24].

**Table 3:** *In vitro* antibacterial activity of OBPgp279, PVP-SE1gp146 and 201ϕ2-1gp229 without (A) and with (B) the OM permeabilizer EDTA against different Gram-negative species, compared to *P. aeruginosa* phage endolysins KZ144 and EL188.

A	OBPgp279	201ϕ2-1gp229	PVP-SE1gp146	KZ144	El188
***P. aeruginosa*** ** PAO1**	1.10±0.07	0.17±0.10	0.19±0.01	0.55±0.14	0.32±0.06
***P. aeruginosa*** ** Br667**	1.08±0.08	0.08±0.03	0.10±0.03	0.34±0.07	0.11±0.04
***E. coli*** ** XL1-Blue**	0.38±0.03	0.04±0.04	0.14±0.01	0.14±0.03	0.21±0.12
***S.*** ** Typhimurium LT2**	0.08±0.01	0.07±0.04	0.13±0.02	0.12±0.04	0.09±0.02

B	EDTA	OBPgp279	201ϕ2-1gp229	PVP-SE1gp146	KZ144	El188
		EDTA	EDTA	EDTA	EDTA	EDTA
***P. aeruginosa*** ** PAO1**	0.58±0.02	4.36±0.29	3.25±0.10	3.68±0,13	3.05±0.11	3.30±0.12
***P. aeruginosa*** ** Br667**	0.03±0.02	3.14±0.22	0.17±0.20	0.12±0,02	0.88±0.04	0.75±0.12
***E. coli*** ** XL1-Blue**	0.29±0.11	1.18±0.20	0.58±0.25	0.34±0,02	0.45±0.13	0.59±0.17
***S.*** ** Typhimurium LT2**	0.05±0.05	0.77±0.05	0.26±0.02	0.14±0,21	0.33±0.14	0.24±0.15

Cell cultures (initial cell density of 10^6^ cells/ml) were incubated for 30 min with 1.5 µM OBPgp279, 5 µM PVP-SE1gp146, 3 µM 201ϕ2-1gp229, 3 µM EL188 and 3 µM KZ144 alone (a) and in combination with 0.5 mM EDTA (b). Antibacterial reduction is calculated in logarithmic units ( = log_10_(N_0_/N_i_) with N_0_ = number of untreated cells and N_i_ = number of treated cells counted after incubation). Averages and standard deviations of three repeated and independent experiments are shown.

The addition of OM permeabilizer EDTA (Table 3B) caused a significant increase in antibacterial activity of OBPgp279 on all four strains. This observation was more pronounced for *P. aeruginosa* PAO1 (3 additional log units) and *P. aeruginosa* Br667 (2 additional log units) than for *E. coli* XL1-Blue and *S.* Typhimurium LT2 (both 0.70 additional log units). For endolysins PVP-SE1gp146 and 201ϕ2-1gp229, only *P. aeruginosa* PAO1 was efficiently reduced in presence of EDTA.

Remarkably, OBPgp279 reduced *P. aeruginosa* PAO1 and the multi-drug resistant *P. aeruginosa* Br667 with 1.10±0.07 and 1.08±0.08 log units, respectively, in the absence of OM destabilizing agents. Contrarily, *E. coli* XL1-Blue and *S.* Typhimurium LT2 showed no sensitivity to the endolysins action. Both PVP-SE1gp146 or 201ϕ2-1gp229 did not influence the two *P. aeruginosa* strains, neither did KZ144 and EL188 (Table 3A).

## Discussion

### Modularity feature in endolysins of Gram-negative origin

Based on their structural and biochemical characteristics, OBPgp279, PVP-SE1gp146 and 201ϕ2-1gp229 are assumed to play an important role in the bacterial host lysis at the end of the phage replication cycle. *In silico*, these three endolysins were shown to share a modular structure with a PBD at the N-terminal side of the protein and a catalytic domain at the C-terminal side. For OBPgp279 and PVP-SE1gp146, the presence of the PBD was experimentally confirmed. This modular composition is rather rare among endolysins with Gram-negative background, as the majority of these endolysins are globular with only a catalytic domain [25]. Even though they share the same general function and modular composition, OBPgp279, PVP-SE1gp146 and 201ϕ2-1gp229 are unrelated at the sequence level. This observation emphasizes the relevance of the biochemical and antibacterial characterization of these enzymes in order to discover new and desirable traits for use as bio-control tools.

We may question why some endolysins of Gram-negative infecting phages have a PBD contained within their structure. For Gram-positive infecting phages, the presence of a PBD is speculated to be necessary to keep the enzyme bound to the cell wall debris. In this way, surrounding bacteria (which are potential candidates for a new infection) remain protected from the hydrolyzing activity of newly released endolysin [25]. In this respect, Loessner and coworkers [26] demonstrated that the *Listeria monocytogenes* phage endolysins Ply118 and Ply500 have a binding affinity (3–6×10^8^ M^−1^) which is comparable with the antigen-ligand affinity in a secondary immune response and can be considered as irreversible. Contrarily, Gram-negative bacteria possess an OM surrounding their PG layer, which eliminates the risk of premature lysis before a new infection can occur. The binding affinities of the Gram-negative phage endolysins KZ144 (2.95×10^7^ M^−1^) [10] and PVP-SE1gp146 (1.26×10^6^ M^−1^) are 10 to 100 times lower but are still relatively high: for PVP-SE1gp146 in equilibrium, only 1 in a million molecules is in the unbound state.

Not only endolysins of phiKZ-related phages feature a modular composition [6], but also members of other unrelated and smaller myoviruses as shown here for *S.* Enteritidis phage PVP-SE1 (“rV5-like virus”) and as described in literature for *P. aeruginosa* phage ϕCTX (“P2-like virus”) [27] and *Burkholderia cepacia* phages Bcep781 and Bcep1 (“Bcep789-like viruses”) [28]. Modular endolysins are assumed to exert a higher enzymatic activity than non-modular or globular ones due to the presence of an additional PG binding domain which keeps the enzyme in close proximity to the PG substrate [25]. This assumption is confirmed here: the muralytic activity of the modular endolysins OBPgp279, PVP-SE1gp146 and 201ϕ2-1gp229 is 160, 104 and 30 times higher compared to the value for the globular phage T4 lysozyme [29]. For an unknown reason, some unrelated phages possess strong and fast-acting modular endolysins instead of less active, globular ones.

### Biochemical activity of modular endolysins

Based on their biochemical activity, OBPgp279 and PVP-SE1gp146 degrade the PG layer of the tested Gram-negative species with the highest efficiencies compared to 201ϕ2-1gp229, EL188 and KZ144. In contrast to the catalytic transglycosylase specificity of KZ144 and EL188 [6], [30], OBPgp279, PVP-SE1gp146 and 201ϕ2-1gp229 are predicted to have lysozyme-like activity. Despite the similar catalytic function, a large variation in muralytic activity values is observed for the individual catalytic domains of OBPgp279 and 201ϕ2-1gp229, due to differences in the primary protein structure. This variation only partially explains the large differences in muralytic activity values of the full length endolysins, since the PBD part also influences the muralytic activity in a protein dependent manner.

Previously, Briers and co-workers [6] revealed the insensitivity of Gram-positive bacteria for the muralytic action of modular endolysins KZ144 and EL188. We found that also OBPgp279, PVP-SE1gp146 and 201ϕ2-1gp229 leave the Gram-positive bacteria *S. aureus* subsp. *aureus* Rosenbach ATCC 6538, *M. lysodeikticus* ATCC 4698, *L. lactis* subsp. *lactis* and *B. subtilis* PSB3 unharmed. An explanation must be found in the structure of their PG layer. Sensitive Gram-negative species share a common A1γ chemotype of PG with a direct peptide bond between adjacent muropeptides, whereas insensitive *S. aureus* subsp. *aureus* Rosenbach ATCC 6538, *M. lysodeikticus* ATCC 4698 and *L. lactis* subsp. *lactis* have a PG of the A3α, A2α and A4α chemotype, respectively [31], [32]. These last 3 chemotypes show resistance for the mode of action of muralytic enzymes, like HEWL, due to the absence of a direct inter-peptide cross-link [33]. *Bacillus subtilis* PSB3, however, possesses the A1γ chemotype of its Gram-negative counterparts but 17.3% of the glucosamine sugars in its PG are N-deacetylated [34]. As previously shown for commercial HEWL, this species-specific modification explains for the resistance against PG lytic enzymes [33]. Therefore, a direct cross-linked A1γ chemotype of PG without substantial N-deacetylation is an essential prerequisite for efficient hydrolysis by endolysins from Gram-negative infecting phages.

### Thermoresistance of PVP-SE1gp146

An unexpected result is the observed thermoresistance of endolysin PVP-SE1gp146 at temperatures up to 90°C. To our knowledge, PVP-SE1gp146 is the first Gram-negative phage derived endolysin described so far to show thermoresistance. For the structural lysin gp36 of *P. aeruginosa* phage ϕKMV (KMV36C) only the catalytic domain-containing C-terminus was thermoresistant [35]. Heat-inactivated, unfolded KMV36C has the opportunity to refold back again into its (partially) active form after cooling and therefore retains its complete enzymatic activity. Recently, this mechanism was also suggested by Schmelcher and coworkers [36] to explain for the high thermostabilty of Gram-positive *Listeria monocytogenes* phage endolysins HPL118 and HPL511. In their study, HPL118 and HPL511 retained 35% of activity after 30 min incubation at 90°C. The authors prove that the unfolding of HPL118 and HPL511 at higher temperatures is followed by a rapid refolding upon cooling. This proposed refolding mechanism may preserve the enzyme activity and could also explain the thermoresistance of PVP-SE1gp146. From this perspective, PVP-SE1gp146 could be an interesting candidate as antibacterial component in a hurdle approach for food preservation, in addition to other antibacterial additives like bacteriocin and nisin [37]. Because of the antibacterial and thermoresistant properties of PVP-SE1gp146, the use of chemical preservatives and the intensity of heat treatments could be reduced in such a way that quality foods with higher nutritional values are obtained.

### Potential use of OBPgp279 as an enzybiotic

Despite the presence of an OM which shields the PG layer from the externally added endolysin, OBPgp279 is capable to reduce both *P. aeruginosa* PAO1 and the multi-drug resistant *P. aeruginosa* Br667, contrary to PVP-SE1gp146, 201ϕ2-1gp229, KZ144 and EL188. Therefore, OBPgp279 is a potential candidate for the use as enzybiotic to control multi-drug resistant opportunistic *Pseudomonas* infections in human and animals. In addition to its high muralytic activity, OBPgp279 is suggested to intrinsically destabilize the OM in the *Pseudomonas* cell wall so that the endolysin reaches its enzymatic target, which could explain the observed reductions. A similar characteristic was suggested for the *Bacillus amyloliquefaciens* phage IAM1521 endolysin *Lys1521* [38]. In the case of this endolysin, the presence of positively charged amino acids in the C-terminal cell wall binding part that interact with the OM and destabilize it, explained the antibacterial property of the endolysin [39]. For OBPgp279, a similar mechanism is unlikely to induce the proposed OM destabilization as its C-terminal end does not contain more positively charged amino acids compared to PVP-SE1gp146 and 201ϕ2-1gp229, which do not affect the tested *Pseudomonas* strains. Furthermore, no obvious amphipathic helixes which are able to intercalate into the OM structure resulting in OM permeabilization [40] were detected at the N- and C-terminal ends of OBPgp279. Further structural analysis of the interaction between OBPgp279 and the *Pseudomonas* cell wall will be necessary to reveal the mechanism and determinants responsible for the observed *Pseudomonas* sensitivity.

### Combinatorial endolysin/EDTA approach

To allow for a better passage of endolysins through the OM a combinatorial approach consisting of an endolysin with an OM permeabilizer was used. In this respect, Briers and coworkers [24] recently demonstrated the efficacy of a combination of an endolysin with Gram-negative background (i.e. endolysin EL188 of *P. aeruginosa* infecting phage EL) and the OM permeabilizer EDTA for antibacterial reduction of the Gram-negative pathogen *P. aeruginosa*. The positive effect of EDTA on antibacterial activity is caused by the powerful OM permeabilization capacity of EDTA through binding and withdrawal of the stabilizing divalent Mg^2+^ and Ca^2+^ cations present in the LPS layer of the Gram-negative OM [20]. As a result, the bacterial PG becomes more prone to the muralytic activity of the externally added endolysins. As with KZ144 and EL188, the three tested modular endolysins induce efficient reduction of the *P. aeruginosa* PAO1 when applied in combination with EDTA. In addition, the OBPgp279/EDTA combination also affects the multidrug-resistant *P. aeruginosa* Br667 strain [8], hinting at the applicability of this combinatorial strategy towards multidrug-resistant *P. aeruginosa* strains as alternative for antibiotic treatment. However, due to the low amount of phosphate groups per LPS molecule and the corresponding amount of stabilizing divalent cations in the OM of *Enterobacteriaceae* compared to that of *Pseudomonas* species [41], the sensitivity of *E. coli* and *S.* Typhimurium strains for the destabilizing EDTA action is much lower. A similar observation was made for endolysin EL188 combined with EDTA [24]. Consequently, the type and structure of the OM seems of crucial importance for the antibacterial efficiency of the endolysin/EDTA combinatorial approach. However, the elucidation of the unique biochemical and antibacterial characteristics of OBPgp279, PVP-SE1gp146 and 201ϕ2-1gp229 (modularity, thermoresistance, OM destabilizing activity) allows us to gain a better insight in the relationship between the structure and activity of endolysins, in general. In this way, we take an important first step in development of future antibacterial therapies based on modular Gram-negative endolysins.

## Supporting Information

Figure S1 **Amino acid sequences of OBPgp279, PVP-SE1gp146 and 201ϕ2-1gp229.** In each sequence the amino acids comprising the PG binding domains are shaded in light grey and the amino acids of the catalytic domains in dark grey. The PG binding motifs which show some similarity with consensus sequence D-G-(Pho)_2_-G-K/N-G/N-T (Pho = hydrophobic amino acid) of the PBDs in KZ144 and EL188 [6] are underlined and marked in bold. A seven amino acid motif L-A-X-Pho-X-L-Y (X = the same hydrophilic amino acid, Pho = hydrophobic amino acid, boxed) is present in front of both repeated PG binding motifs of OBPgp279, each with four amino acids in between. Inside the catalytic domains of OBPgp279 and PVP-SE1gp146, the detected motifs showing similarity to the general consensus sequence for GH19 family members (F/H/Y-G-R-G-A/P-X-Q-I/L-S/T-F/H/Y/W-H/N-F/Y-N/Y, X = hydrophilic amino acid) [16] are underlined in italic.
(TIF)

Figure S2 **Bacterial host spectrum of OBPgp279 (light grey bars), PVP-SE1gp146 (intermediate grey bars) and 201ϕ2-1gp229 (dark grey bars).** Each endolysin (1 µM final concentration) is added to outer membrane permeabilized *P. aeruginosa* PAO1 (PA), *S.* Typhimurium LT2 (ST), *E. coli* XL1-Blue (EC) and intact *S. aureus* subsp. *aureus* Rosenbach ATCC 6538 (SA), *M. lysodeikticus* ATCC 4698 (ML), *B. subtilis* PSB3 (BS) and *L. lactis* subsp. *lactis* (LL). The resulting decrease of OD_655 nm_ in function of time after endolysin addition is depicted here. Averages and standard deviations of three independent experiments are given.
(TIF)

Figure S3 **pH optimization for enzymatic activity of OBPgp279 (diamonds), PVP-SE1gp146 (squares) and 201ϕ2-1gp229 (triangles).** Muralytic activity is measured as the slope of the ΔOD_655 nm_/min curve (Y-axis) on OM permeabilized *P. aeruginosa* PAO1 substrate and is shown for a pH range between 3 and 10 (X-axis). Final concentrations used here are 1 µM OBPgp279, 5 µM PVP-SE1gp146 and 3 µM 201ϕ2-1gp229, each dialyzed against a PBS buffer (pH 7.4). Averages and standard deviations of three repeated experiments are given.
(TIF)

Table S1 Overview of all expression constructs used in this study. The specific insert, cloning vector, primers, expression/purification conditions and expression yield are indicated for each construct. The restriction endonuclease recognition sites inside the primers are underlined. Extra nucleotides necessary for in frame cloning are indicated in bold. All constructs were expressed using the *E. coli* BL21(DE3)pLysS expression strain in LB medium upon induction with 1 mM IPTG. Proteins were purified on Ni^2+^- NTA columns using protein dependent imidazole concentrations given in the table. Expression yield is indicated in mg of obtained recombinant protein per liter expression culture as determined spectrophotometrically.
(DOCX)
