# Supplementary figures and images for: Characterization of Modular Bacteriophage Endolysins from Myoviridae Phages OBP, 201ϕ2-1 and PVP-SE1
Source: PLoS One. 2012 May 15;7(5):e36991. doi: 10.1371/journal.pone.0036991 (PMC3352856; doi:10.1371/journal.pone.0036991)

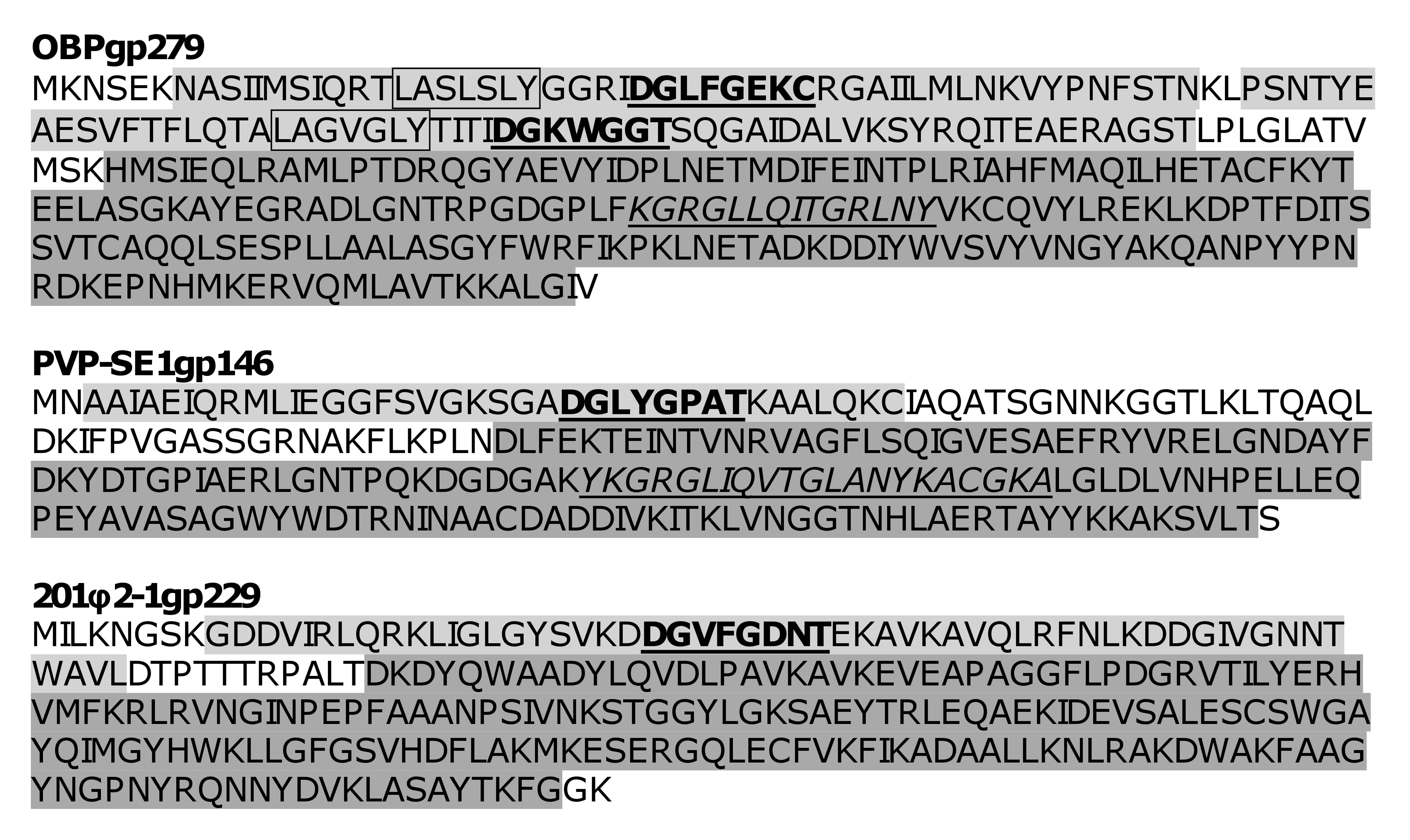

Supplement: Figure S1 — Amino acid sequences of OBPgp279, PVP-SE1gp146 and 201ϕ2-1gp229. In each sequence the amino acids comprising the PG binding domains are shaded in light grey and the amino acids of the catalytic domains in dark grey. The PG binding motifs which show some similarity with consensus sequence D-G-(Pho)2-G-K/N-G/N-T (Pho = hydrophobic amino acid) of the PBDs in KZ144 and EL188 [6] are underlined and marked in bold. A seven amino acid motif L-A-X-Pho-X-L-Y (X = the same hydrophilic amino acid, Pho = hydrophobic amino acid, boxed) is present in front of both repeated PG binding motifs of OBPgp279, each with four amino acids in between. Inside the catalytic domains of OBPgp279 and PVP-SE1gp146, the detected motifs showing similarity to the general consensus sequence for GH19 family members (F/H/Y-G-R-G-A/P-X-Q-I/L-S/T-F/H/Y/W-H/N-F/Y-N/Y, X = hydrophilic amino acid) [16] are underlined in italic. (TIF) [file pone.0036991.s001.tif]

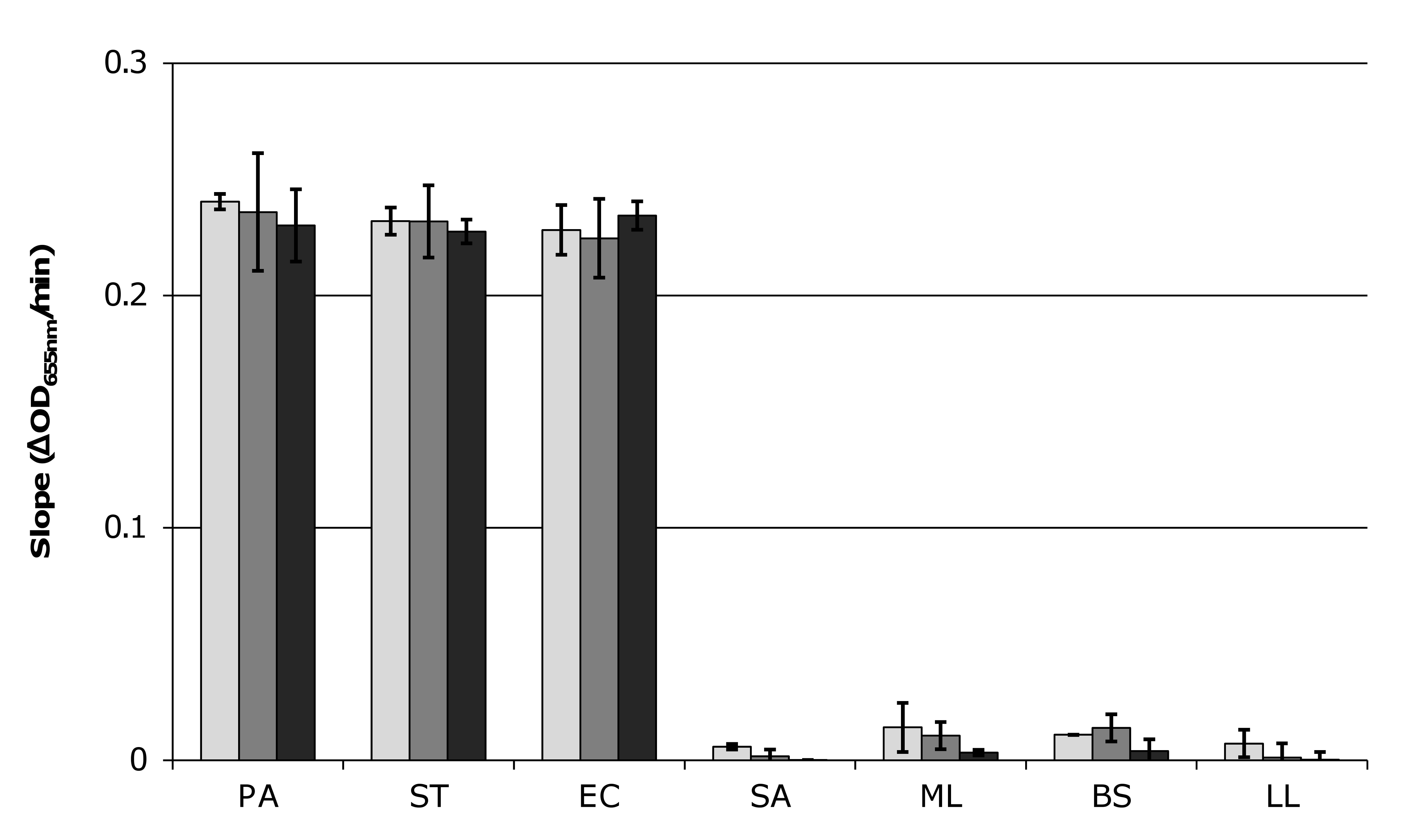

Supplement: Figure S2 — Bacterial host spectrum of OBPgp279 (light grey bars), PVP-SE1gp146 (intermediate grey bars) and 201ϕ2-1gp229 (dark grey bars). Each endolysin (1 µM final concentration) is added to outer membrane permeabilized P. aeruginosa PAO1 (PA), S. Typhimurium LT2 (ST), E. coli XL1-Blue (EC) and intact S. aureus subsp. aureus Rosenbach ATCC 6538 (SA), M. lysodeikticus ATCC 4698 (ML), B. subtilis PSB3 (BS) and L. lactis subsp. lactis (LL). The resulting decrease of OD655 nm in function of time after endolysin addition is depicted here. Averages and standard deviations of three independent experiments are given. (TIF) [file pone.0036991.s002.tif]

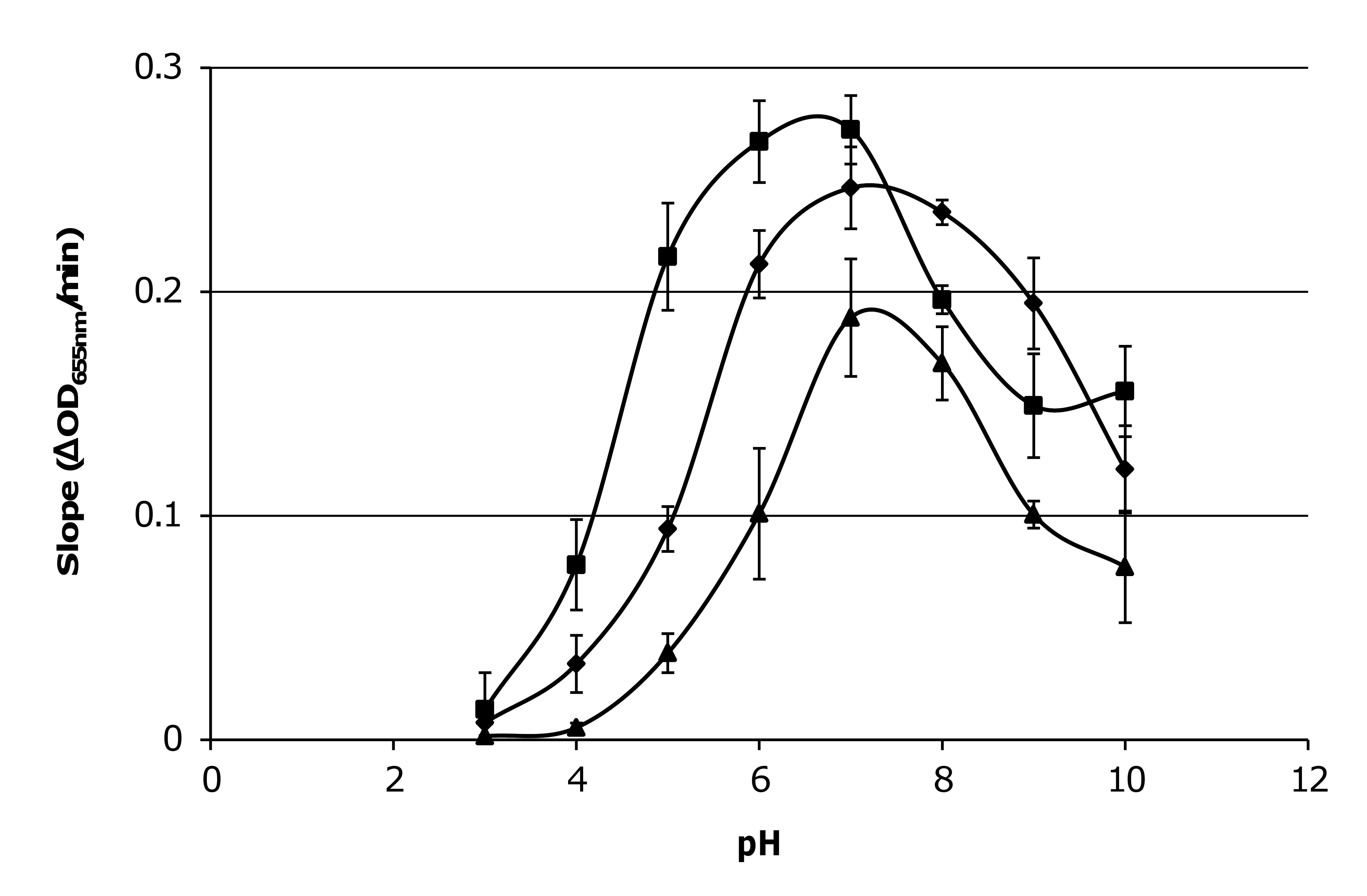

Supplement: Figure S3 — pH optimization for enzymatic activity of OBPgp279 (diamonds), PVP-SE1gp146 (squares) and 201ϕ2-1gp229 (triangles). Muralytic activity is measured as the slope of the ΔOD655 nm/min curve (Y-axis) on OM permeabilized P. aeruginosa PAO1 substrate and is shown for a pH range between 3 and 10 (X-axis). Final concentrations used here are 1 µM OBPgp279, 5 µM PVP-SE1gp146 and 3 µM 201ϕ2-1gp229, each dialyzed against a PBS buffer (pH 7.4). Averages and standard deviations of three repeated experiments are given. (TIF) [file pone.0036991.s003.tif]
