# Supplementary material for: Characterization of Modular Bacteriophage Endolysins from Myoviridae Phages OBP, 201ϕ2-1 and PVP-SE1
Source: PLoS One. 2012 May 15;7(5):e36991. doi: 10.1371/journal.pone.0036991 (PMC3352856; doi:10.1371/journal.pone.0036991)
Supplement: Table S1 — Overview of all expression constructs used in this study. The specific insert, cloning vector, primers, expression/purification conditions and expression yield are indicated for each construct. The restriction endonuclease recognition sites inside the primers are underlined. Extra nucleotides necessary for in frame cloning are indicated in bold. All constructs were expressed using the E. coli BL21(DE3)pLysS expression strain in LB medium upon induction with 1 mM IPTG. Proteins were purified on Ni2+- NTA columns using protein dependent imidazole concentrations given in the table. Expression yield is indicated in mg of obtained recombinant protein per liter expression culture as determined spectrophotometrically. (DOCX) [file pone.0036991.s004.docx]

| **Insert encoding for** | **Vector for ligation** | **Primers** | **Expression and purification conditions** | **Yield (in mg/l culture)** |
| --- | --- | --- | --- | --- |
| **OBPgp279** | pEXP5CT-TOPO® | ATGAAAAATAGCGAGAAGAAT AACTATTCCGTGTGCTTTCTTTGT | 37°C/4h/65 mM imidazole | 3.30 |
| **PVP-SE1gp146** | pEXP5CT-TOPO® | ATGGGATCCAATGCTGCAATTGCGGAGA CGAGGTTAGAACAGATTTTGCCT | 16°C/18h/70 mM imidazole | 38.18 |
| **201φ2-1gp229** | pEXP5CT-TOPO® | ATGGGATCCATCCTTAAAAACGGCTC CTTTCCACCGAACTTTG | 37°C/4h/65 mM imidazole | 18.92 |
| **OBP_127-327_** | pEXP5CT-TOPO® | ATGGGATCCCATATGTCTATTGAACAG TATTCCGAGTGCTTTCT | 37°C/4h/60 mM imidazole | 24.63 |
| **PVP_82-235_** | pEXP5CT-TOPO® | ATGGGATCCGACCTGTTTGAAAAG TAGAACAGATTTTGCC | 37°C/4h or 16°C/18h and 50 mM imidazole | No soluble protein |
| **201φ2-1_75-260_** | pEXP5CT-TOPO® | ATGGGATCCGATAAAGATTATCAATGGGCTG ACCGAACTTTGTATACGCGCT | 37°C/4h/60 mM imidazole | 2.27 |
| **OBP_1-117_-EGFP** | pEGFP – *Pst*I/*Bam*HI pEXP5CT-TOPO® | GGCCTGCAG**T**AAAAATAGCGAGAAG TTAGGATCC**AT**CACAGTAGCAAGACCTAAT | 37°C/4h/60 mM imidazole | 2.60 |
| **OBP_7-54_-EGFP** | pEGFP – *Pst*I/*Bam*HI pEXP5CT-TOPO® | GGCCCTGCAG**T**AATGCATCGATAATTATGTC CGTGGATCC**AT**GTTGGTGCTGAAATTG | 37°C/4h/ 60 mM imidazole | 0.17 |
| **OBP_57-117_-EGFP** | pEGFP - *Pst*I/*Bam*HI pEXP5CT-TOPO® | GCGCTGCAG**T**CCGAGTAACACATATGA CGTGGATCC**AT**CAACGTCGACCCAGC | 37°C/4h/60 mM imidazole | 0.08 |
| **PVP_1-63_-EGFP** | pEGFP – *Pst*I/*Bam*HI pEXP5CT-TOPO® | GGCCTGCAG**T**AATGCTGCAATTG TTAGGATCC**AT**TTTGTCCAGTTGTGC | 16°C/18h/65 mM imidazole | 8.71 |
| **201φ2-1_8-63_-EGFP** | pEGFP – *Pst*I/*Bam*HI pEXP5CT-TOPO® | GGCCTGCAG**T**GGCGATGACGTTATTCGTCT GTCGGATCCATCAATACAGCCCATGTGTTATT | 37°C/4h/60 mM imidazole | 2.54 |

**Table S1. Overview of all expression constructs used in this study.**
